# Eco-friendly preparation of titanium dioxide/carbon nitride nanocomposites for photoelectrocatalytic applications

**DOI:** 10.1039/d5na00478k

**Published:** 2025-08-05

**Authors:** Hanna Maltanava, Nikita Belko, Konstantin Tamarov, Niko M. Kinnunen, Pauliina Nevalainen, Martynas Zalieckas, Renata Karpicz, Igor Koshevoy, Dmitry Semenov, Sari Suvanto, Sergei Malykhin, Vesa-Pekka Lehto, Polina Kuzhir

**Affiliations:** a Department of Physics and Mathematics, University of Eastern Finland Joensuu Finland polina.kuzhir@uef.fi; b Department of Technical Physics, University of Eastern Finland Kuopio Finland; c Department of Chemistry and Sustainable Technology, University of Eastern Finland Joensuu Finland; d Department of Molecular Compound Physics, Center for Physical Sciences and Technology Vilnius Lithuania; e School of Computing, University of Eastern Finland Joensuu Finland

## Abstract

Titanium dioxide (TiO_2_) and its heterostructures are among the most extensively studied materials for photo- and electrocatalytic applications. Optimizing their synthesis remains crucial for enhancing performance and reducing production costs. In this work, we report a simple, eco-friendly method for preparing TiO_2_/graphitic carbon nitride (g-C_3_N_4_) nanocomposites in both powder and thin-film forms. The method takes advantage of the catalytic properties of TiO_2_ to significantly lower the temperature required for the formation of g-C_3_N_4_ from urea, from 600 °C to 300 °C. Incorporating lyophilization prior to thermal treatment results in a *ca*. 60% increase in the specific surface area. The materials were evaluated for their photo- and electrocatalytic performance. Upon photoactivation at 385 nm, both TiO_2_ and TiO_2_/g-C_3_N_4_ powders generate the hydroxyl radical, with lyophilization enhancing radical production fivefold. The lyophilized TiO_2_/g-C_3_N_4_ nanocomposite exhibits 14% higher photocatalytic activity than its TiO_2_ counterpart. In electrocatalytic studies, TiO_2_/g-C_3_N_4_ thin films demonstrate a 70 mV lower overpotential for oxygen reduction compared to TiO_2_ films. These results highlight the potential of the synthesized nanocomposites for environmental remediation and in energy-related applications such as fuel cell electrodes.

## Abstract 2

Titanium dioxide/graphitic carbon nitride nanocomposites exhibit efficient photocatalytic generation of the hydroxyl radical and improved oxygen electroreduction.

## Graphical abstract

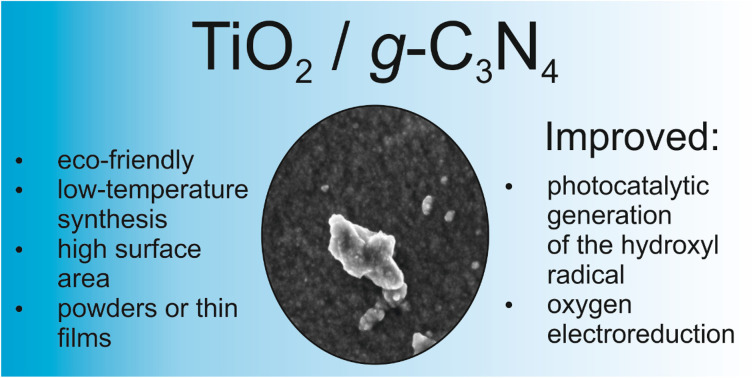

## 1 Introduction

Photocatalytic oxidation reactions that occur in semiconductor materials have attracted significant attention as an eco-friendly solution to environmental pollution.^1^ The mechanism of photocatalytic oxidation typically involves the generation of reactive species, such as superoxide radical anion (˙O_2_^−^), hydrogen peroxide (H_2_O_2_), singlet oxygen (^1^O_2_), and hydroxyl radical (˙OH), which can efficiently mineralize organic pollutants.^2–4^ Although numerous new photoactive materials have been reported, TiO_2_ has been considered as one of the most popular photocatalysts due to its chemical inertness, strong oxidizing power, nontoxicity, and long-term stability against photocorrosion.^5–10^ Although the photocatalytic activity of TiO_2_ depends on various structural and surface characteristics, titania with a large surface area and a high degree of porosity is often required to achieve high efficiency in photocatalytic applications.^11,12^ Mesoporous TiO_2_ powders and films exhibit a high surface area and a narrow pore size distribution while retaining a crystalline framework.^6–9,11,13–16^ Furthermore, the mesoporous structure enhances the diffusion of reactants and products while also improving the access to the reactive sites on the surface of a photocatalyst.^17,18^ Further research is still being conducted with the aim of identifying mesoporous catalytic materials with enhanced characteristics.

The activity of TiO_2_ in photoreactions critically depends on the generation of the OH radical, one of the strongest oxidizing agents.^19^ In particular, the generation of the free OH radical (˙OH_f_) and its subsequent diffusion from the surface of the catalyst are essential in achieving the decomposition of non-adsorbing substrates by extending the reaction from the surface to the solution bulk. The photocatalytic generation of ˙OH_f_ by TiO_2_ strongly depends on the kind of crystal polymorph. More specifically, anatase efficiently produces ˙OH_f_, while OH radicals generated by rutile mostly remain adsorbed on its surface.^20,21^ The photocatalytic generation of ˙OH_f_ by anatase can proceed *via* the reductive 3e^−^ pathway (O_2_ → ˙O_2_^−^ → H_2_O_2_ → ˙OH_f_) or the oxidative process (OH^−^ + h^+^ → ˙OH_f_).^20^ The reaction mechanism is still under debate and requires additional studies.^21^

To date, various strategies have been implemented to improve the performance of TiO_2_ photocatalysts including doping with metal and non-metal elements, surface sensitization, the creation of semiconductor heterojunctions, *etc*.^22,23^ Among these methods, heterostructuring TiO_2_ with other semiconductor materials is one of the most effective methods to inhibit the fast electron–hole recombination in pure TiO_2_,^24^ thus enhancing the photocatalytic performance. Ideally, heterostructures should be designed in such a way as to tailor the generation of reactive intermediates and free radicals to a particular application.

Carbon nitrides, in general, and graphitic carbon nitride (g-C_3_N_4_), in particular, are frequently selected as the preferred materials for heterostructuring TiO_2_.^25,26^ g-C_3_N_4_ has been reported to be non-toxic, environmentally friendly, and stable.^27,28^ It has been used for a variety of photocatalytic applications, including water splitting,^29,30^ H_2_ and O_2_ evolution,^31,32^ CO_2_ reduction,^29,30^ H_2_O_2_ production,^33,34^ pollutant degradation,^30,31^ and selective oxidation of organic compounds.^31^

TiO_2_ and g-C_3_N_4_ are often chosen for the preparation of heterostructures, because the edges of both the valence and conduction bands of g-C_3_N_4_ have higher energy values with respect to TiO_2_. Such an electronic structure of the resulting heterostructure is conducive of efficient charge separation, with the electrons preferably accumulating in the conduction band of TiO_2_ and the holes favoring the valence band of g-C_3_N_4_. Indeed, in previous works, composites of carbon nitrides with TiO_2_ were successfully applied as photocatalytic materials, *e.g.*, for CO_2_ photoreduction,^35^ photocatalytic decomposition of N_2_O,^36^ NH_3_ production,^37^ H_2_ evolution,^38^ photocatalytic degradation of organic dyes,^39^ and photocurrent generation.^40^ In ref. 41, carbon nitride–TiO_2_ hybrid was shown to outperform its components in several photo- and photoelectrocatalytic reactions while exhibiting enhanced activity in the visible range.^41^ Enhanced generation of ˙OH by carbon nitride–TiO_2_ heterostructures can be expected as well.

A widely used approach to the synthesis of carbon nitride–TiO_2_ hybrid materials is to prepare the two phases separately and then use them to create a composite.^35,36,42–44^

In this work, we prepare TiO_2_/g-C_3_N_4_ powders and thin films from TiO_2_ sol using a different procedure. A precursor of g-C_3_N_4_ (urea or melamine) is added directly to TiO_2_ sol prior to thermal treatment. The TiO_2_ nanoparticles are shown to catalyze the polymerization of urea to g-C_3_N_4_ at a much lower temperature (300 °C *vs.* 600 °C for pure urea) resulting in a simple and eco-friendly synthesis. We demonstrate the effect of the g-C_3_N_4_ precursor (urea *vs.* melamine), lyophilization, and the annealing temperature on the properties of the resulting materials. The prepared samples are characterized using X-ray diffractometry (XRD), X-ray photoelectron spectroscopy (XPS), scanning electron microscopy (SEM), transmission electron microscopy (TEM), and diffuse reflectance spectroscopy (DRS). The specific surface area and porosity of the prepared materials are measured. Finally, the photocatalytic activity in ˙OH production and the electrocatalytic activity in the oxygen reduction reaction (ORR) are tested.

## 2 Experimental

### 2.1 Materials

TiCl_4_ (99.9%), HCl (37%), HNO_3_ (70%), ammonia solution (25%), urea (>99.5%), KOH (>90%), and terephthalic acid (>98%) were purchased from Merck. Melamine (>98.5%) was purchased from Thermo Scientific. All solutions were prepared in deionized (DI) water.

### 2.2 Synthesis of TiO_2_/g-C_3_N_4_ nanocomposites

**Fig. 1:**
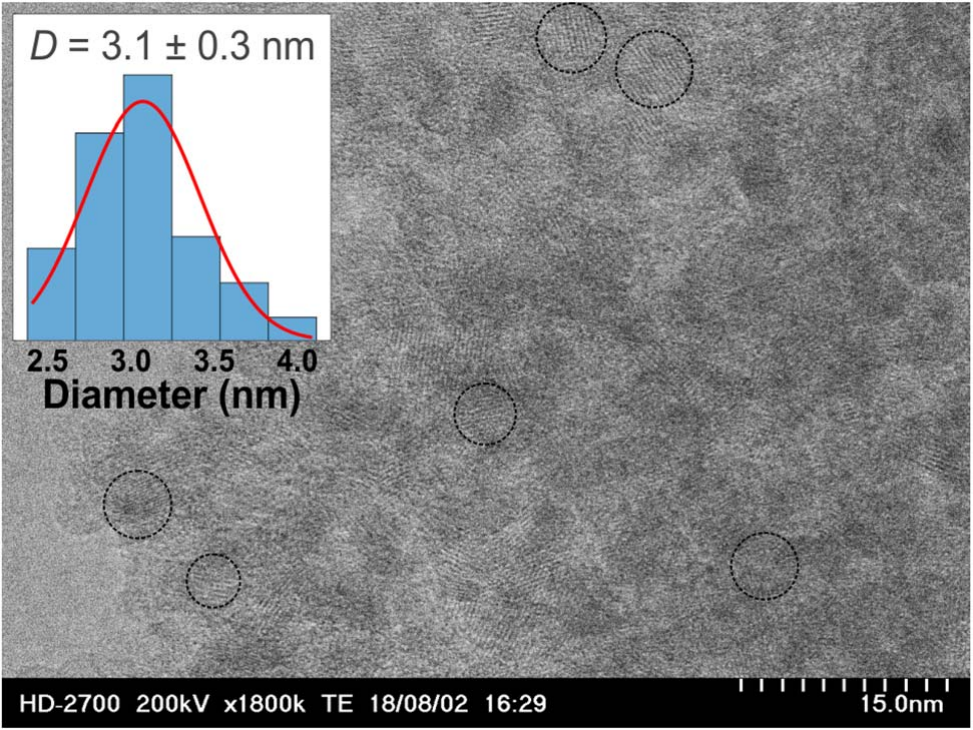
TEM micrograph of TiO_2_ nanoparticles drop-cast from the sol. Several typical particles are marked with dashed circles. The inset shows the corresponding size distribution (blue bars) and the fitting Gaussian function (red curve).

First, a concentrated TiO_2_ sol was prepared using the sol–gel technique. The following operations (up to centrifugation) were performed in an ice bath, and the reagents were cooled to 0 °C before use. To prepare the sol, 15 mL of TiCl_4_ was added dropwise to 53 mL of 0.65 M aqueous HCl under vigorous stirring. The resulting clear yellowish solution was diluted to 250 mL with DI water and titrated with a 12% aqueous ammonia solution with vigorous stirring. As the pH reached 4–5, the suspension became viscous and the titration and stirring were stopped. The suspension was then centrifuged at 5000 rpm for 5 min. The supernatant was discarded, 250 mL DI water was added, and the precipitate was resuspended. Centrifugation and washing with DI water was repeated 4 times. The supernatant was discarded, and 1.6 mL concentrated HNO_3_ (65 wt%) was added to the washed precipitate to promote peptization. The resulting suspension was sonicated for 10 min with an ultrasonic horn (22 kHz). As the suspension was heated by sonication, it became a transparent thick gel. The gel was cooled until it became liquid again. The steps of sonication and cooling of the suspension were repeated three times. Finally, the sol was centrifuged at 5000 rpm for 15 min, and the sediment was discarded. The TiO_2_ content in the sol was determined by weight analysis after drying 3 mL of the sol at 800 °C. The resulting transparent opalescent sol contained 12 wt% TiO_2_ and remained stable for several months at room temperature. A similar synthetic procedure was previously reported in ref. 45. As evidenced by TEM, the sizes of the TiO_2_ nanoparticles in the formed sol were 3.1 ± 0.3 nm (Fig. 1).

In ref. 46, the properties of g-C_3_N_4_ were shown to depend on the choice of precursor (urea *vs.* melamine). Here, we attempted to polymerize both urea and melamine in the presence of the prepared TiO_2_ nanoparticles to obtain the g-C_3_N_4_ phase. 5 mL of the TiO_2_ sol (corresponding to 0.6 g TiO_2_) was mixed with 0.6 g of urea or melamine. Note that mixing of the sol with melamine resulted in coagulation, while this effect was not observed with urea. The resulting suspensions containing TiO_2_ nanoparticles and urea/melamine were placed in a ceramic crucible with a cover and subjected to thermal treatment at 200, 300, 450, or 600 °C for 2 h. The heating rate was 2 °C min^−1^. The neat TiO_2_ sol was also subjected to similar thermal treatment. The resulting powders were thoroughly milled with agate mortar and pestle.

We also attempted to improve the synthetic procedure by including the lyophilization step. More precisely, 1.5 mL of the TiO_2_ sol was mixed with 0.4 g urea, and the mixture was lyophilized. The resulting powder was then placed in a ceramic crucible with a cover and subjected to thermal treatment at 200 or 300 °C for 2 h. The heating rate was 2 °C min^−1^. The neat TiO_2_ sol was also subjected to similar processing. The resulting powders were thoroughly milled with agate mortar and pestle.

In the following, samples are denoted as (L)-TiO_2_(/g-C_3_N_4_)-T, where “L” indicates that the sample was lyophilized prior to thermal treatment and T is the temperature of the synthesis. For instance, L-TiO_2_/g-C_3_N_4_-300 refers to a TiO_2_/g-C_3_N_4_ nanocomposite that was lyophilized and then subjected to thermal treatment at 300 °C.

A similar procedure was also implemented to prepare thin films of TiO_2_ and TiO_2_/g-C_3_N_4_. 5 mL TiO_2_ sol was mixed with 0.6 g urea, and the resulting mixture (as well as the neat TiO_2_ sol) was spin coated onto FTO-coated glass. The samples were then subjected to thermal treatment at 300 °C for 2 h. The heating rate was 2 °C min^−1^.

**Fig. 2:**
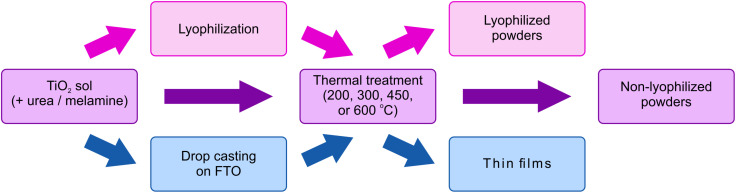
The synthetic procedure for the preparation of TiO_2_ and TiO_2_/g-C_3_N_4_ in the form of non-lyophilized powders, lyophilized powders, and thin films.

The synthetic procedure described in this section is summarized in Fig. 2.

Pure g-C_3_N_4_ was prepared by placing pure urea in a ceramic crucible with a cover and subjecting it to thermal treatment at 600 °C for 2 h. The heating rate was 2 °C min^−1^.

### 2.3 Characterization techniques

#### 2.3.1 X-ray diffraction

XRD patterns were measured with a PANalytical Empyrean diffractometer using CuKα-radiation. The recording speed was 0.4° min^−1^. The interplanar distance *d* was calculated from the XRD peak positions using Bragg's law (eqn (1)):

1 2*d* sin *θ* = *nλ*,

where *θ* is the glancing angle, *n* is the diffraction order, and *λ* = 1.54 Å is the wavelength of CuKα-radiation. The mean crystallite size *τ* was estimated using Debye–Scherrer equation (eqn (2)):2
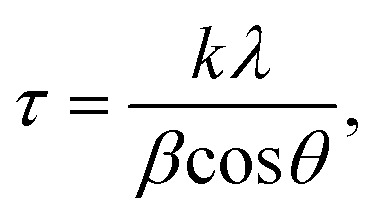
where *k* = 0.9 is the shape factor for spheroid particles and *β* is the full width at half maximum of the XRD peak used for the calculations.

#### 2.3.2 X-ray photoelectron spectroscopy

The powdered samples were measured in a Cu powder holder using a Nexsa G2 spectrometer (Thermofisher Scientific Inc.). The measurements were done with a 400 μm X-ray spot size (Al Kα-radiation) and concurrent use of an electron flood gun operating in charge compensation mode. The measurements were started with a 1-min delay to equilibrate possible transient effects. For all samples, low-resolution survey spectra were first collected (pass energy of 200 eV, step size of 1 eV, dwell time of 10 ms), followed by an automatic identification of the present elements. After that, the selected peaks were measured in high-resolution mode (pass energy of 50 eV, step size of 0.1 eV, dwell time of 50 ms). The high-resolution spectra were then used for elemental content analysis and peak fitting for chemical state analysis using Avantage v6.9 software (Thermo Fisher Scientific Inc.). For the analysis, the peaks were first aligned to Ti 2p_3/2_ of 458.8 eV since slight charging was observed despite the use of the flood gun. Peak fitting was performed using the smart background with varied peak components, each consisting of a Gaussian–Lorentzian product mixture (30% Lorentzian). Sensitivity factors accounting for variations in photoionization cross-sections were automatically applied during elemental quantification, utilizing values retrieved from the Avantage software database.

#### 2.3.3 Electron microscopy

The size of TiO_2_ nanoparticles was determined using a Hitachi HD2700D transmission electron microscope. The morphology of the prepared samples was studied using a Zeiss LEO 1550 scanning electron microscope in the secondary electron detection mode. The accelerating voltage was 3 kV for the images acquired at a magnification of 50k× and 10 kV for the high-resolution images acquired at a magnification of 500k×.

#### 2.3.4 Gas adsorption measurements

Specific surface area measurements were performed with a Microtrac Belsorp MAX X device. Before measurement, a sample was pretreated at 150 °C for 2 h. N_2_ gas physical adsorption was carried out at the temperature of liquified nitrogen. After physical adsorption of N_2_ gas, its desorption was recorded as well. Specific surface areas were obtained from the physical adsorption data by applying Brunauer–Emmett–Teller (BET) theory. To achieve average pore size distributions of the materials in the microporous range, the micropore analysis method (MP-method)^47,48^ was applied.

#### 2.3.5 Diffuse reflectance spectroscopy

DRS spectra were measured using a PerkinElmer Lambda 1050 spectrophotometer equipped with a 150 mm InGaAs integrating sphere.

### 2.4 Photocatalytic activity

The photocatalytic activity of the prepared materials was studied using a light-emitting diode (LED) peaking at 385 nm with a bandwidth (FWHM) of 11 nm (CHROLIS, Thorlabs). The light was coupled into a 3 mm liquid light guide and passed through a collimating adapter (SLSLLG3, Thorlabs) to achieve a collimated light beam. The middle part of the light beam (1 × 1 cm^2^) illuminated samples inside a 1 cm quartz cuvette at normal incidence. The injection current of the LED was adjusted so that the irradiance at the front face of the cuvette was 60 mW cm^−2^. Prior to irradiation, the samples were bubbled with O_2_ gas for 10 min. During irradiation, O_2_ bubbling was continued accompanied by agitation with a magnetic stirrer.

˙OH production was registered using terephthalic acid (TA) as a fluorogenic probe. The method was adopted from ref. 49 with modifications. TA exhibits no fluorescence under 310 nm excitation. After oxidation by ˙OH, 2-hydroxyterephthalic acid (HTA) is produced (Fig. S1). Under 310 nm excitation, HTA emits fluorescence peaked at 425 nm. The emission intensity at 425 nm was regarded as the parameter reflecting ˙OH production. TA was dissolved in 0.1 M KOH and then diluted 10-fold with DI water resulting in a solution containing 3 mM TA and 10 mM KOH. The prepared TiO_2_/g-C_3_N_4_ and TiO_2_ powders were dispersed in the TA solution with sonication (5 min) at a concentration of 1 mg mL^−1^ and were then agitated with a magnetic stirrer for 15 min prior to the irradiation. After irradiation, the samples were centrifuged at 14 000 rpm (19 000*g*) for 15 min. Finally, the supernatants were collected, and their fluorescence spectra were registered under 310 nm excitation using an Edinburgh FLS1000 spectrofluorimeter (2 × 1.5 nm excitation and emission slits).

### 2.5 Electrocatalytic activity

Electrocatalytic activity with respect to the oxygen reduction reaction (ORR) was examined using cyclic voltammetry for TiO_2_/g-C_3_N_4_ and TiO_2_ thin films prepared on FTO-coated glass slides. Cyclic voltammograms were acquired using an Autolab PGSTAT 302N potentiostat/galvanostat at a potential scan rate of 10 mV s^−1^. The measurements were conducted in a single-compartment glass cell equipped with three electrodes. The TiO_2_/g-C_3_N_4_ or TiO_2_ samples served as the working electrode. The area of the working electrode was 1 cm^2^. The reference electrode was an Hg/HgO electrode filled with 1 M KOH, and the counter electrode was a Pt foil. The potential of the reference electrode was 115 mV *vs.* a saturated calomel electrode. The supporting electrolyte was 0.1 M aqueous KOH (pH 13) saturated with O_2_ gas for 1 h prior to the measurements.

## 3 Results and discussion

### 3.1 Characterization of TiO_2_/g-C_3_N_4_ nanocomposites

The thermal treatment temperatures for the studied materials were selected to fulfill two primary objectives. First, to ensure the formation of the anatase phase of TiO_2_, which exhibits superior catalytic activity compared to the rutile phase, particularly in the generation of hydroxyl radicals (˙OH_f_).^20,21^ Second, to facilitate the conversion of precursor materials (melamine or urea) to the target g-C_3_N_4_ phase.

**Fig. 3:**
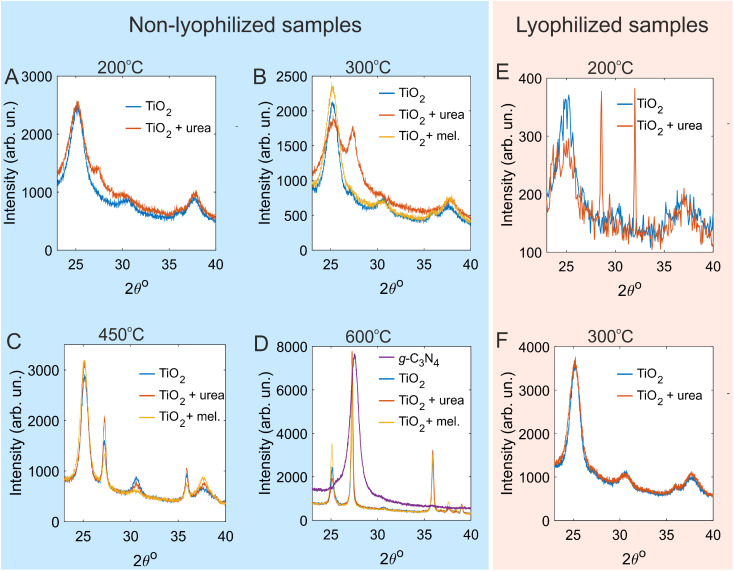
XRD patterns for non-lyophilized (A)–(D) and lyophilized (E) and (F) samples of TiO_2_ and TiO_2_/g-C_3_N_4_. (A)–(D). The TiO_2_ sol (blue curves), the TiO_2_ sol mixed with urea (red curves), and the TiO_2_ sol mixed with melamine (yellow curves) that were subjected to thermal treatment at 200 °C (A), 300 °C (B), 450 °C (C), and 600 °C (D). Panel D also shows the XRD pattern for pure urea polymerized at 600 °C to yield g-C_3_N_4_ (purple curve). (E) and (F). The TiO_2_ sol (blue curves) and the TiO_2_ sol mixed with urea (red curves) that were lyophilized and then subjected to thermal treatment at 200 °C (E) and 300 °C (F).

The prepared materials were examined using XRD to establish the optimal parameters for synthesis. TiO_2_ powder subjected to thermal treatment at 200 or 300 °C consisted of anatase (JCPDS no. 21-1272) with a small admixture of brookite (JCPDS no. 29-1360) (Fig. 3A and B). The mean size of anatase crystallites calculated from the half-width of the (101) diffraction peak (25.5° (ref. 50)) using the Debye–Scherrer equation was 7 nm for the samples annealed at 200 °C and rose to 9 nm after annealing at 300 °C. In the XRD pattern for TiO_2_ annealed at 450 °C, the narrowing of the anatase reflexes and the appearance of rutile reflexes were evident (Fig. 3C). The XRD pattern for TiO_2_ annealed at 600 °C was dominated by narrow peaks of rutile (for instance, the (110) peak at 27.3° (ref. 50)), [JCPDS no. 21-1276], but weak and narrow anatase peaks were still present (Fig. 3D). According to the XRD data, thermal treatment of TiO_2_ at 450 or 600 °C resulted in an increase in the crystallinity and the transformation of anatase to rutile. These observations are in line with previously reported data indicating that the anatase-to-rutile transformation occurs in the 400–1200 °C temperature range.^50^

The influence of the temperature and the choice of the g-C_3_N_4_ precursor (urea *vs.* melamine) was then studied for the samples that were not subjected to lyophilization prior to thermal treatment. The XRD pattern for the TiO_2_ sol mixed with urea and annealed at 200 °C contained a broad weak peak at 27.4° in addition to the TiO_2_ reflexes (Fig. 3A). For the TiO_2_ sol mixed with urea and annealed at 300 °C, a pronounced peak at 27.4° was observed (Fig. 3B). These peaks can be identified as the (002) reflex of g-C_3_N_4_.^46,51^ In contrast, when melamine was used instead of urea, no g-C_3_N_4_ phase was formed. This could be due to the coagulation of the TiO_2_ sol after the introduction of melamine. The TiO_2_ sol mixed with urea or melamine and heated to 450 °C exhibited XRD patterns almost identical to that of the pure TiO_2_ sol subjected to the same treatment (Fig. 3C). This finding indicates that urea and melamine were completely decomposed, and g-C_3_N_4_ was not formed. Similarly, the g-C_3_N_4_ phase was not observed after annealing at 600 °C (Fig. 3D). For comparison, the polymerization of pure urea at 600 °C resulted in the formation of highly crystalline g-C_3_N_4_ (Fig. 3D). This is evidenced by the presence of a strong and narrow band peaked at 27.6° corresponding to the (002) reflex of tri-*s*-triazine based g-C_3_N_4_.^46,51^ The corresponding interplanar distance (calculated using Bragg's law) was found to be 0.33 nm, consistent with the interlayer spacing in tri-*s*-triazine based g-C_3_N_4_.^46,51^ Note that the (110) reflex of rutile and the (002) reflex of g-C_3_N_4_ have close peak positions (27.3° and 27.6°, respectively). They can still be distinguished as the (002) reflex of g-C_3_N_4_ is slightly shifted to larger 2*θ* values and is substantially wider (Fig. 3D).

As follows from the XRD analysis of the prepared materials, the samples prepared from the TiO_2_ sol and urea at 200 °C and 300 °C had a two-phase composition of TiO_2_ (anatase) and g-C_3_N_4_. For the sample prepared at 200 °C, the amount of the g-C_3_N_4_ phase was smaller and/or the g-C_3_N_4_ phase was poorly crystallized. Thermal treatment of urea in the presence of TiO_2_ at 450 or 600 °C resulted in a complete decomposition of urea. The use of melamine did not allow for the formation of g-C_3_N_4_ at any temperature. The formation of g-C_3_N_4_ from pure urea was achieved only at 600 °C. These data seem to indicate that the TiO_2_ nanoparticles efficiently catalyze the polymerization of urea and enable the synthesis of the g-C_3_N_4_ phase at substantially lower temperatures (200–300 °C). At higher temperatures, the presence of TiO_2_ causes urea to decompose. Thus, further characterization is performed for samples TiO_2_(/g-C_3_N_4_)-200 and TiO_2_(/g-C_3_N_4_)-300 prepared from the TiO_2_ sol and urea.

The influence of lyophilization on the crystallinity and the phase composition of the resulting materials was also studied. The XRD patterns for the lyophilized TiO_2_ sol annealed at 200 or 300 °C (Fig. 3E and F) indicated that the samples consisted of anatase. For TiO_2_ that was mixed with urea, lyophilized, and then annealed at 200 °C (sample L-TiO_2_/g-C_3_N_4_-200), no reflexes of g-C_3_N_4_ were observed (Fig. 3E). Instead, narrow peaks at 16.8, 22.7, 28.6, 32.0, and 53.4° were present (see also Fig. S2). The peak at 22.7° can be identified as the most intense reflex of urea (although slightly shifted, which could be due to low intensity and the influence of TiO_2_). The rest of the peaks could be ascribed to the products of urea transformations. These data indicate that the thermal treatment of lyophilized powders at 200 °C was not sufficient to fully polymerize urea. After annealing at 300 °C (Fig. 3F), no residual XRD peaks of urea or intermediates were observed. Reflexes of g-C_3_N_4_ were not evident, too. The absence of reflexes of g-C_3_N_4_ in lyophilized samples might indicate that the crystallites were not sufficiently large to appear in XRD patterns or that the g-C_3_N_4_ phase formed was amorphous.^41^

Elemental composition of all samples was determined using XPS (Table S1). XPS characterization was performed for the non-lyophilized TiO_2_ and TiO_2_/g-C_3_N_4_ samples prepared at 200 and 300 °C to analyze their bonding configurations. As shown in Fig. S3, all samples showed the presence of carbon (C 1s), nitrogen (N 1s), titanium (Ti 2p) and oxygen (O 1s). The ratio of Ti and O abundances was very close to 1 : 2 for all samples, and the formation of composites did not seem to affect this ratio (Table S1). For sample TiO_2_/g-C_3_N_4_-300, the ratio of C and N abundances was close to the expected stoichiometry of 3 : 4. The Ti 2p spectra of the TiO_2_/g-C_3_N_4_ composites have two peaks at 458.8 and 464.5 eV, corresponding to Ti 2p_3/2_ and Ti 2p_1/2_, respectively. These positions with the peak separation of 5.7 eV indicate octahedrally coordinated Ti^4+^ in TiO_2_.^52–54^ The O 1s peak located at 530.1 eV is assigned to lattice oxygen in TiO_2_.^36,42^ Samples TiO_2_/g-C_3_N_4_-200 and TiO_2_/g-C_3_N_4_-300 also exhibit a noticeable O 1s peak at 531–532 eV, which most likely belongs to the surface hydroxyl groups or organic C–O and C=O bonds formed during thermal polymerization of urea.^55^

The C 1s spectrum for sample TiO_2_/g-C_3_N_4_-300 was fitted with three peaks located at binding energies of 288.1, 285.5, and 289.4 eV, which correspond to sp^2^ carbon, C–C/C=C, and COOH bands, respectively.^36,56,57^ In the N 1s region, the main components were observed at 398.8 and 399.7 eV, corresponding to the sp^2^-hybridized N atoms in the heptazine rings and tertiary N atoms bonded to carbon atoms in the form of N–(C)_3_ or H–N–(C)_2_, respectively.^42,57,58^ The N 1 s peak at 398.2 eV is related to weak C–N or N–N bonds linked to sp^2^ carbon.^59^ The XPS data confirm the existence of graphite-like sp^2^ bonded structure in graphitic carbon nitride. In addition to the three described N 1s peaks, the contribution at 406.4 eV can be assigned to the –NO_2_ groups on the surface of TiO_2_ (ref. 60) (which are present due to nitric acid in the TiO_2_ sol).

The XPS spectra for sample TiO_2_/g-C_3_N_4_-200 are presented in Fig. S3. The C 1s signal reveals three components, namely 285.9, 289.2, and 292.2 eV. The peak with the lowest binding energy is associated with C atoms with sp^3^ diamond bonds and does not belong to pure g-C_3_N_4_. It may be assigned to terminal C–NH_*x*_ groups^61^ or C–C/C=C bonds.^62^ The C 1s signal at 289.2 eV can be attributed to the overlap of signals from sp^2^ carbon, carboxylic groups, and sp^2^ carbon in the aromatic ring attached to –NH_2_ groups.^63,64^ In addition, a weak signal at 292.2 eV in the C 1s spectrum can be assigned to the π electron delocalization in g-C_3_N_4_ heterocycles, confirming graphitic stacking of triazine- or heptazine-based layers.^65^ The lowest energy contribution of the N 1s spectrum, 399.2 eV, is attributed to nitrogen bonded with two carbon atoms in a graphitic sp^2^ network.^66^ The peak at 400.4 eV corresponds to the overlap of signals from bridging nitrogen atoms, such as tertiary N (N–(C)_3_), and amino groups (–NH_*x*_), revealing the presence of tri-*s*-triazine rings.^63^ The presence of –N–C=O band was also identified in the N 1s spectrum (the peak at 401.5 eV).^67^ The N 1s peak at 407.5 eV can be attributed to nitrate groups on the oxide surface.^68^

XPS analysis was also performed for the lyophilized TiO_2_ and TiO_2_/g-C_3_N_4_ samples prepared at 200 and 300 °C (Fig. S4). All samples demonstrated Ti 2p and O 1s spectra similar to those of their non-lyophilized counterparts. The ratio of Ti and O abundances remained almost equal to 1 : 2 (Table S1). At the same time, the carbon content was similar for samples L-TiO_2_-300 and L-TiO_2_/g-C_3_N_4_-300 due to the presence of adventitious carbon. The C 1s spectra for all lyophilized samples demonstrated three bands peaking at 284.7, 286.5, and 289.2 eV, which were ascribed to C–C, C–N/C=C, and sp^2^-hybridized carbon in the triazine ring attached to NH_2_ species (N=C–N_2_), respectively.^69–71^ The spectrum for the sample prepared at 200 °C was dominated by the non-graphitic C–C bonds (284.7 eV), whereas, the contribution from sp^2^ carbon (289.2 eV) was the most intense band in the spectrum for the sample prepared at 300 °C. Although pure TiO_2_ and TiO_2_/g-C_3_N_4_ composites demonstrated similar C 1s peaks, the contribution from sp^2^-hybridized carbon at 289.2 eV was higher for the composites (Fig. S4).

The N 1s spectrum for sample L-TiO_2_/g-C_3_N_4_-300 revealed bands at 398.1 and 400.1 eV, which were assigned to sp^2^ nitrogen and tertiary nitrogen (N–(C)_3_), respectively. The N 1s spectrum for sample L-TiO_2_/g-C_3_N_4_-200 was characterized by two components at 399.3 and 400.3 eV. The peak at 399.3 eV corresponded to the overlap of signals from sp^2^-hybridized imine groups (C–N=C) and surface functional amino groups (–NH_*x*_).^72,73^ The highest energy contribution at 400.3 eV can be assigned to tertiary nitrogen groups (N–(C)_3_). Abundance of N in the composites was substantially higher compared to pure TiO_2_ (Table S1). Taken together, changes in the C 1s contributions and the increase in the abundance of N in the composites suggest that a small amount of the g-C_3_N_4_ phase was formed.

Based on the results of the XRD and XPS analyses, it was confirmed that the prepared composites (both non-lyophilized and lyophilized) annealed at 300 °C contained both the TiO_2_ and g-C_3_N_4_ phases. At the same time, the composites prepared at 200 °C contained the anatase and g-C_3_N_4_ phases as well as urea residues and/or products of its thermal decomposition. The XPS data for L-TiO_2_/g-C_3_N_4_ composites suggest the formation of N-defective nanocrystalline g-C_3_N_4_.^74^ However, g-C_3_N_4_ in the sample prepared at 200 °C was poorly polymerized, and residual urea and intermediates were still present.

**Fig. 4:**
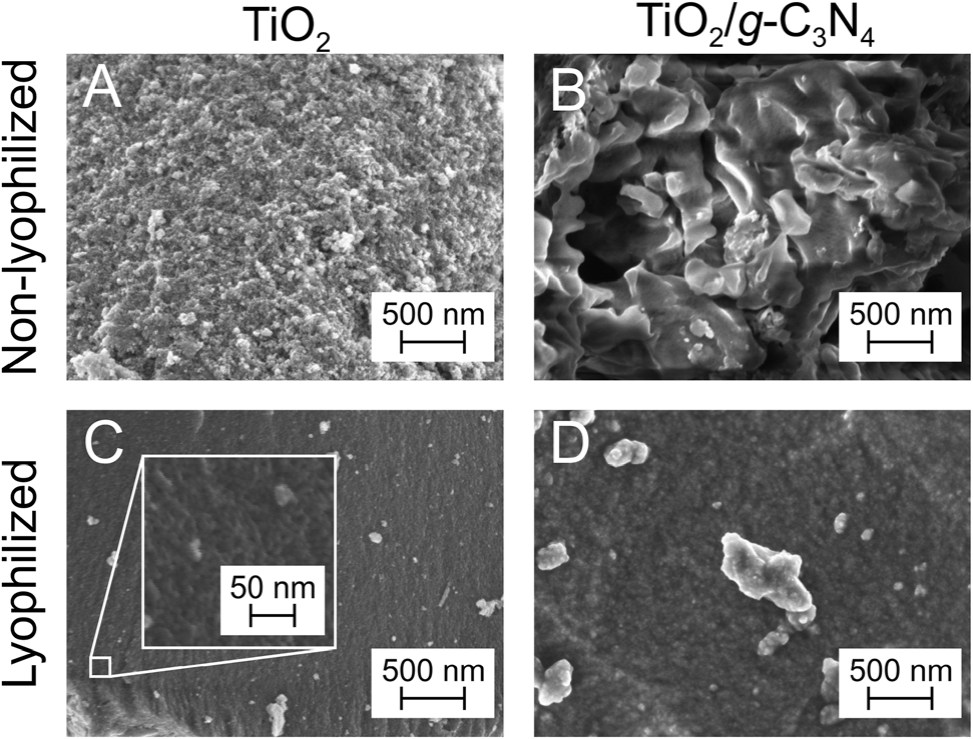
SEM micrographs for TiO_2_ (A) and (C) and TiO_2_/g-C_3_N_4_ (B) and (D) samples not subjected (A) and (B) and subjected (C) and (D) to lyophilization before thermal treatment at 300 °C. The images were acquired at a magnification of 50k×. The inset in panel C shows a high-resolution image acquired at a magnification of 500k× to resolve the nanoporous structure of this sample.

The morphology of non-lyophilized and lyophilized TiO_2_ and TiO_2_/g-C_3_N_4_ powders was studied using SEM (Fig. 4). Sample TiO_2_-300 exhibited porous structure with a characteristic particle size of *ca*. 100 nm. For sample TiO_2_/g-C_3_N_4_-300, much larger, micron-sized features were observed. These can be identified as g-C_3_N_4_ crystals, in line with XRD and XPS data discussed above. Sample L-TiO_2_-300 consisted of particles that were *ca*. 10 nm large, which is consistent with the particle size in the TiO_2_ sol as determined by TEM (Fig. 1). Sample L-TiO_2_/g-C_3_N_4_-300 was characterized by a slightly larger particle size. Thus, TiO_2_ nanoparticles in non-lyophilized samples were prone to sintering. Furthermore, adding urea to the TiO_2_ sol and subjecting this liquid mixture to thermal treatment seemed to promote the growth of the g-C_3_N_4_ crystalline phase on the surface of TiO_2_. Lyophilization not only prevented the sintering of TiO_2_ nanoparticles but also inhibited the growth of large g-C_3_N_4_ crystals.

SEM data were further corroborated by N_2_ physisorption analysis. The lyophilized samples obtained at 200 and 300 °C exhibited different types of the N_2_ adsorption–desorption isotherms. As can be seen from Fig. S5, according to the IUPAC classification,^75^ the type I isotherm is observed for the lyophilized samples annealed at 200 °C. This type of isotherm represents the physical adsorption process on microporous adsorbents. However, there are differences in BET surface areas and micropore size distributions–urea modification decreased both. The specific surface area, as calculated by the multi-point BET method, was found to be 185 and 137 m^2^ g^−1^, respectively. The decrease in the specific surface area for L-TiO_2_/g-C_3_N_4_-200 can be explained by pore-blocking caused by products of poor polymerization of urea. Samples L-TiO_2_-300 and L-TiO_2_/g-C_3_N_4_-300 exhibited the type IV isotherm with an H2a hysteresis loop at *P*/*P*_0_ of 0.4–0.7 (Fig. S6), which is characteristic of mesoporous solids having pores with a uniform size distribution.^76^ The specific surface area was 108 m^2^ g^−1^ for L-TiO_2_-300 sample, which increased up to 231 m^2^ g^−1^ for L-TiO_2_/g-C_3_N_4_-300 sample, and pore size in the microporous area increased from 1.2 to 1.4 nm after the addition of urea. The non-lyophilized TiO_2_-300 and TiO_2_/g-C_3_N_4_-300 samples had a mesoporous structure (type IV isotherm, H2b hysteresis) with a wide range of sizes in pore restrictions or pore entrances. (Fig. S7). In addition to meso- and micropores, the sample contained a certain amount of macropores, as evidenced by the rise of the isotherm at *P*/*P*_0_ > 0.9. The BET surface area was 132 m^2^ g^−1^ for sample TiO_2_-300 and 140 m^2^ g^−1^ for sample TiO_2_/g-C_3_N_4_-300. The pore sizes of both samples in the microporous range were *ca*. 1.5 nm, although for sample TiO_2_-300, the pore determination was not so clear because the graph has more than one maximum point.

Characterization data for the prepared TiO_2_/g-C_3_N_4_ composites and pure TiO_2_ powders are summarized in Table 1.

**Table 1:** Specific surface area and phase composition of the prepared materials

Sample	Specific surface area (m^2^ g^−1^)	g-C_3_N_4_ state
TiO_2_-300	132	−
TiO_2_/g-C_3_N_4_-300	140	+
L-TiO_2_-200	185	−
L-TiO_2_/g-C_3_N_4_-200	137	+ (as well as urea and intermediates)
L-TiO_2_-300	108	−
L-TiO_2_/g-C_3_N_4_-300	231	+ (nanocrystalline)

By using lyophilization and changing the annealing temperature, TiO_2_/g-C_3_N_4_ composites with different characteristics can be prepared. Based on BET analysis, the non-lyophilized samples exhibited smaller specific surface area. Since annealing at 200 °C was insufficient for the complete polymerization of urea, treatment at 300 °C should be chosen.

**Fig. 5:**
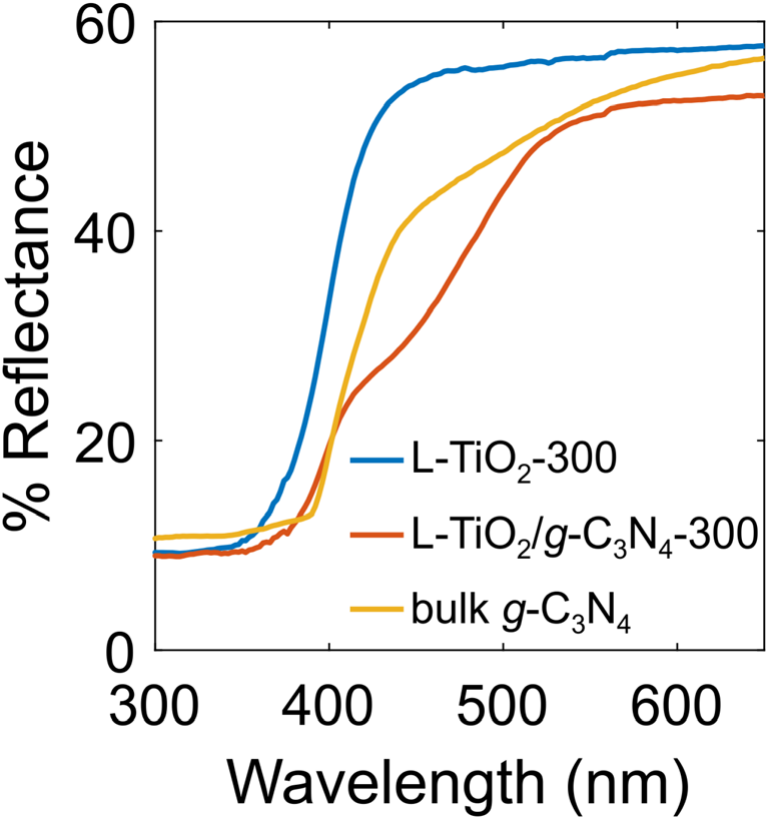
Diffuse reflectance spectra for lyophilized TiO_2_ powder (blue curve) and TiO_2_/g-C_3_N_4_ nanocomposite (red curve) prepared at 300 °C as well as for bulk g-C_3_N_4_ prepared by polymerizing urea at 600 °C (yellow curve).

The optical response of the prepared materials was studied using DRS (Fig. 5). A diffuse reflectance spectrum for the L-TiO_2_/g-C_3_N_4_-300 nanocomposite seems to be a combination of the spectra for the pure g-C_3_N_4_ and TiO_2_ phases.

### 3.2 Photo- and electrocatalytic activity of TiO_2_/g-C_3_N_4_ nanocomposites

The production of the OH radical, one of the strongest oxidizing agents,^19^ is a key parameter of a photocatalyst used for the degradation of chemically resistant organic compounds, including environmental pollutants. Several works explored the photocatalytic ˙OH production by TiO_2_.^20,21^ Anatase was shown to exhibit superior activity compared to rutile in the generation of the free, non-adsorbed OH radical. Since TiO_2_ studied in this work consists of anatase, efficient ˙OH production is expected. The presence of the g-C_3_N_4_ phase could further improve the yield of the OH radical.

**Fig. 6:**
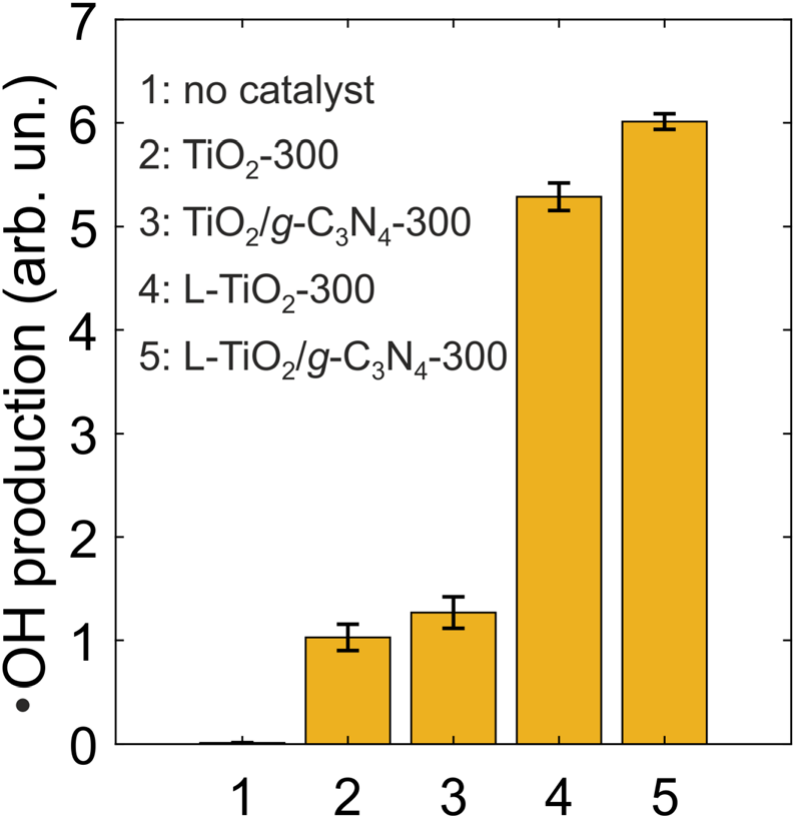
˙OH production (as HTA fluorescence intensity) without catalysts and in 1 mg mL^−1^ suspensions of non-lyophilized and lyophilized TiO_2_ powders and TiO_2_/g-C_3_N_4_ composites annealed at 300 °C. The photoactivation was performed with an LED peaking at 385 nm at a light dose density of 36 J cm^−2^ (60 mW cm^−2^ for 10 min).

Thus, non-lyophilized and lyophilized TiO_2_/g-C_3_N_4_ nanocomposites and TiO_2_ powders were compared with respect to the photocatalytic ˙OH production upon activation with near-UV (385 nm) light. Both samples TiO_2_-300 and TiO_2_/g-C_3_N_4_-300 generated the OH radical, with the nanocomposite being slightly more efficient (Fig. 6). The lyophilized samples L-TiO_2_-300 and L-TiO_2_/g-C_3_N_4_-300 demonstrated a drastic 5-fold improvement in ˙OH production compared to their non-lyophilized counterparts. Between the two lyophilized samples, the nanocomposite containing the g-C_3_N_4_ phase was more efficient by 14% with respect to ˙OH production. The diminished photocatalytic activity observed in the non-lyophilized samples is likely attributable to sintering during thermal treatment and the resulting poor dispersibility in aqueous media. The enhanced ˙OH generation upon incorporation of the g-C_3_N_4_ phase can be ascribed to improved charge separation, facilitated by the favorable alignment of the valence and conduction band edges of the TiO_2_ and g-C_3_N_4_ components.

**Fig. 7:**
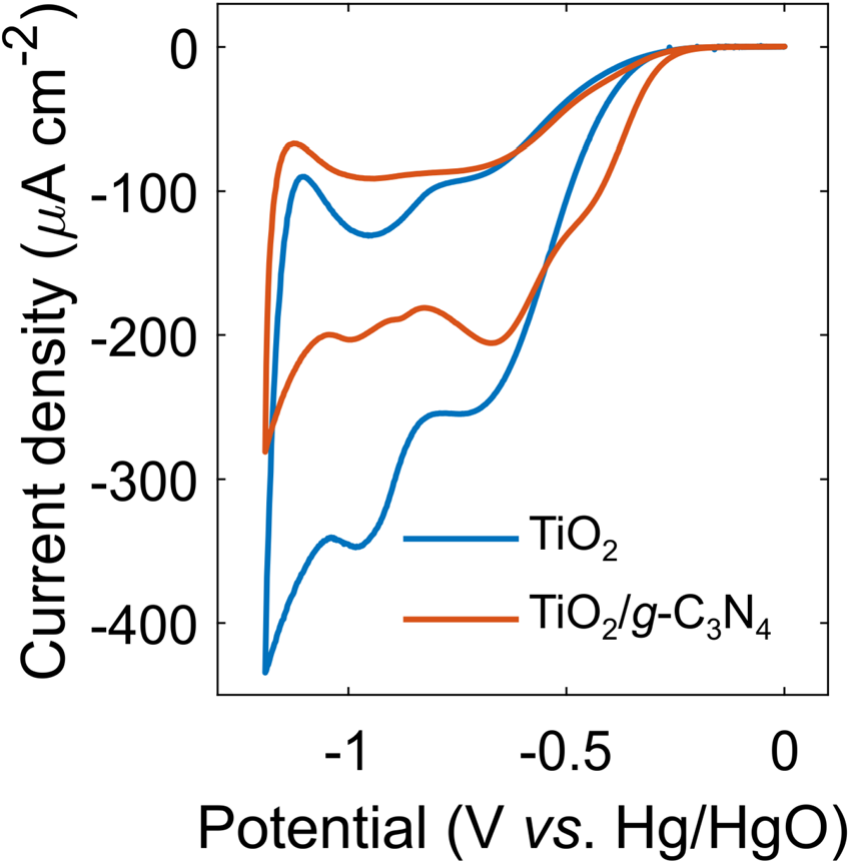
Cyclic voltammograms for the ORR on the TiO_2_ (blue curve) and TiO_2_/g-C_3_N_4_ (red curve) thin films prepared on FTO at 300 °C.

Improving the performance of electrode materials with respect to the ORR is important for the development of more cost-efficient fuel cells. The electrocatalytic activity of TiO_2_ and TiO_2_/g-C_3_N_4_ thin films in the ORR was studied (Fig. 7). The cyclic voltammogram for the TiO_2_ film demonstrated two waves of oxygen electroreduction peaked at −0.74 and −0.98 V *vs.* Hg/HgO (or 0.15 and −0.09 V, respectively, *vs.* reversible hydrogen electrode). For the TiO_2_/g-C_3_N_4_ film, the onset of oxygen electroreduction occurred at a more positive potential. At the current density of −100 μA cm^−2^, the reduction in the ORR overpotential amounted to 70 mV. Heterostructures of hollow carbon nanospheres and graphitic C_3_N_5_ demonstrated a similar reduction in ORR overpotential with respect to bare nanoshperes.^77^ For comparison, loading TiO_2_/carbon black composite with Pt nanoparticles resulted in a decrease in the ORR overpotential by *ca*. 200 mV.^78^ As anticipated, the TiO_2_/g-C_3_N_4_ composite did not match the electrocatalytic performance of platinum-based materials, which serve as standard benchmarks for the ORR. However, reducing reliance on precious metals aligns with the principles of sustainable development. The electrocatalytic activity of TiO_2_/g-C_3_N_4_ can be further enhanced through careful optimization of synthesis parameters or loading it with noble-metal nanoparticles.

Photocatalytic and electrocatalytic properties for the studied materials demontrate similar ímprovements due to the introduction of the g-C_3_N_4_ phase. Photocatalytic ˙OH production and electrocatalytic oxygen reduction share certain material-dependent characteristics, particularly the critical role of O_2_ adsorption. In photocatalysis, the reduction of O_2_ by trapped electrons on TiO_2_ has been identified as the rate-limiting step in the oxidation of organic compounds,^79,80^ whereas in electrocatalysis, O_2_ adsorption significantly influences the overpotential. Despite these similarities, the two processes differ in other performance-determining factors. Enhanced photocatalytic activity can be attributed to improved dispersibility achieved through lyophilization and favorable band alignment that promotes charge separation. In contrast, the reduced overpotential observed in oxygen electroreduction is more likely due to increased electrical conductivity and the greater availability of active sites.

## Conclusions

In this work, TiO_2_/g-C_3_N_4_ nanocomposites were prepared by polymerizing urea in the presence of TiO_2_. Unlike urea, melamine was shown to be unsuitable as a precursor of g-C_3_N_4_ formed in the presence of TiO_2_. For non-lyophilized samples, the g-C_3_N_4_ phase was formed after annealing at 200 or 300 °C. The lyophilization of the TiO_2_ sol or the mixture of the sol with urea prior to annealing resulted in materials with substantially larger specific surface areas. After lyophilization, annealing at 300 °C was required for a complete polymerization of urea. Thus, the material with optimal characteristics was obtained when using urea as the g-C_3_N_4_ precursor, implementing lyophilization, and performing thermal treatment at 300 °C.

Overall, the higher sample processing temperature increased the pore size diameter of the samples and increased the number of mesopores. Urea addition decreased both BET surface area and pore size diameter when temperature of 200 °C was applied while processing temperature of 300 °C increased both BET surface area and pore size diameter. Lyophilization treatment increased BET surface area only if urea was added together with higher processing temperature (300 °C).

Lyophilized TiO_2_ and TiO_2_/g-C_3_N_4_ samples prepared at 300 °C generated the hydroxyl radical 5 times more efficiently compared to their non-lyophilized counterparts upon photoactivation in the near-UV range. Furthermore, the lyophilized TiO_2_/g-C_3_N_4_ composite demonstrated a 14% increase in ˙OH production in comparison to the lyophilized TiO_2_ powder. The TiO_2_/g-C_3_N_4_ thin film prepared at 300 °C showed a 70-mV reduction in the oxygen electroreduction overpotential compared to the bare TiO_2_ thin film.

The described synthetic approach differs from the previously reported procedures, which involved the preparation g-C_3_N_4_ and TiO_2_ separately and then mixing them.^35,36,42–44^ The synthetic procedure described here results in materials with lowered crystallinity of the phases (especially, when lyophilization is implemented), but a substantially larger specific surface area.

The low-temperature conditions required for synthesizing the composites enhance both cost and energy efficiency. Moreover, the process eliminates the need for surfactants, which can be non-biodegradable and potentially harmful to the environment. This eco-friendly synthesis approach aligns with the principles of sustainable materials development while yielding composites with good catalytic performance.

## Author contributions

Hanna Maltanava: conceptualization, data curation, investigation, methodology, visualization, writing – original draft. Nikita Belko: conceptualization, data curation, investigation, methodology, visualization, writing – original draft. Konstantin Tamarov: data curation, investigation, methodology, visualization, writing – review & editing. Niko M. Kinnunen: data curation, investigation, methodology, visualization, writing – original draft. Pauliina Nevalainen: data curation, investigation, methodology, visualization, writing – original draft. Martynas Zalieckas: data curation, investigation, methodology. Renata Karpicz: conceptualization, supervision, writing – review & editing. Igor Koshevoy: funding acquisition, methodology, supervision. Dmitry Semenov: methodology, investigation, supervision. Sari Suvanto: investigation, methodology, resources, supervision. Sergei Malykhin: investigation, methodology. Vesa-Pekka Lehto: funding acquisition, resources, supervision. Polina Kuzhir: conceptualization, funding acquisition, methodology, resources, supervision, writing – review & editing.

## Conflicts of interest

There are no conflicts to declare.

## Supplementary Material

NA-OLF-D5NA00478K-s001

## Data Availability

Data for this article, including XRD patterns, XPS spectra, DRS spectra, SEM images, cyclic voltammograms, and N_2_ adsorption–desorption isotherms are available at Zenodo at https://doi.org/10.5281/zenodo.15267936. Scheme of the oxidation of terephthalic acid, additional XRD curves, element abundances, XPS spectra, N_2_ adsorption–desorption isotherms, and micropore size distributions. See DOI: https://doi.org/10.1039/d5na00478k.
