# Supplementary material for: Eco-friendly preparation of titanium dioxide/carbon nitride nanocomposites for photoelectrocatalytic applications
Source: Nanoscale Adv. 2025 Aug 5;7(18):5601–11. doi: 10.1039/d5na00478k (PMC12322928; doi:10.1039/d5na00478k)
Supplement: NA-007-D5NA00478K-s001 [file NA-007-D5NA00478K-s001.pdf]

Supplementary Information (SI) for Nanoscale Advances.
This journal is © The Royal Society of Chemistry 2025

# *Electronic Supporting Information for*

# Eco-friendly preparation of titanium dioxide / carbon nitride nanocomposites for photoelectrocatalytic applications

Hanna Maltanava, Nikita Belko, Konstantin Tamarov, Niko M. Kinnunen, Pauliina Nevalainen, Martynas Zalieckas, Renata Karpicz, Igor Koshevoy, Dmitri Semenov, Sari Suvanto, Sergei Malykhin, Vesa-Pekka Lehto, Polina Kuzhir

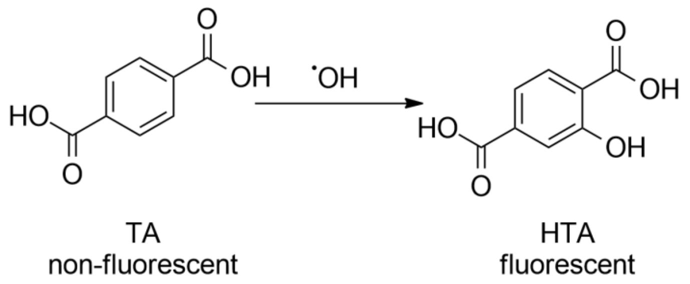


**Fig. S1**. Detection of the OH radical using terephathalic acid (TA). Upon oxidation of non-fluorescent TA by •OH, fluorescent 2-hydroxyterephthalic acid (HTA) is formed with an emission peak at 425 nm.

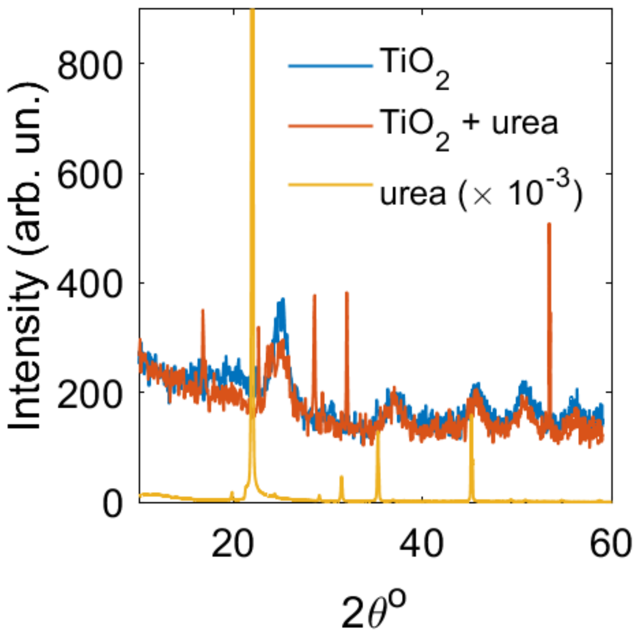


**Fig. S2**. XRD pattern for the $TiO_2$ sol (blue curve) and the $TiO_2$ sol mixed with urea (red curve) that were lyophilized and then subjected to thermal treatment at 200 $^oC$. Yellow curve shows an XRD pattern for pure, untreated urea.

**Table S1**. Elemental composition of the studied $TiO_2$ and $TiO_2$/*g*-$C_3N_4$ samples as determined by XPS.

| **Sample** | **Ti (at.%)** | **O (at.%)** | **C (at.%)** | **N (at.%)** |
|---|---|---|---|---|
| **$TiO_2$-200** | 27.9 | 63.3 | 5.6 | 3.3 |
| **$TiO_2$/*g*-$C_3N_4$-200** | 18.2 | 47.2 | 47.2 | 20.2 |
| **$TiO_2$-300** | 30.4 | 61.3 | 6.2 | 2.1 |
| **$TiO_2$/*g*-$C_3N_4$-300** | 8.0 | 22.2 | 27.1 | 42.8 |
| **L-$TiO_2$-200** | 29.2 | 60.3 | 6.5 | 3.7 |
| **L-$TiO_2$/*g*-$C_3N_4$-200** | 21.3 | 46.6 | 16.0 | 13.7 |
| **L-$TiO_2$-300** | 29.7 | 60.3 | 5.9 | 3.6 |
| **L-$TiO_2$/*g*-$C_3N_4$-300** | 28.0 | 57.2 | 6.1 | 6.2 |

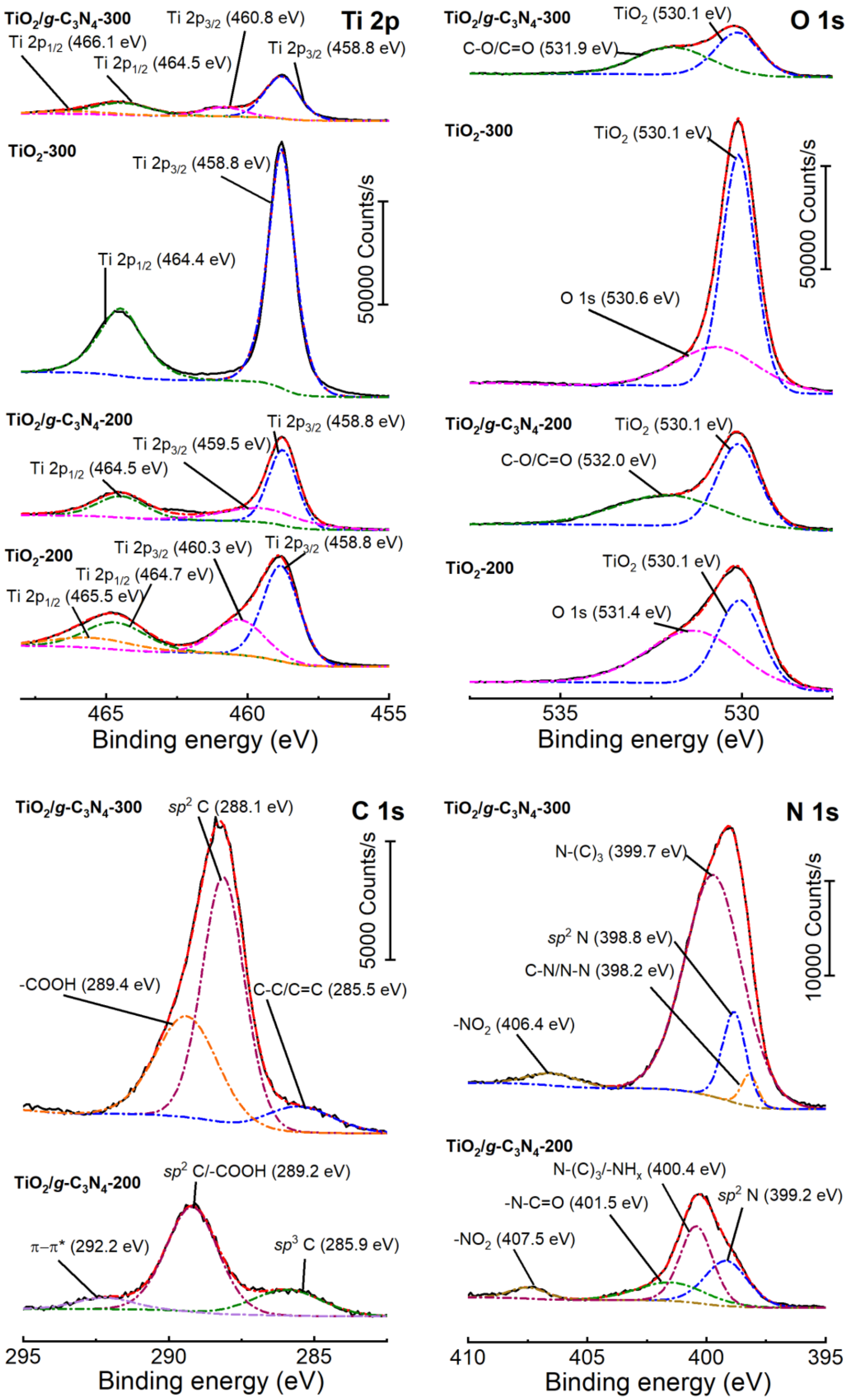


**Fig S3**. High-resolution Ti 2p, O 1s, C 1s, and N 1s spectra for non-lyophilized $TiO_2$ and $TiO_2$/*g*-$C_3N_4$ samples.

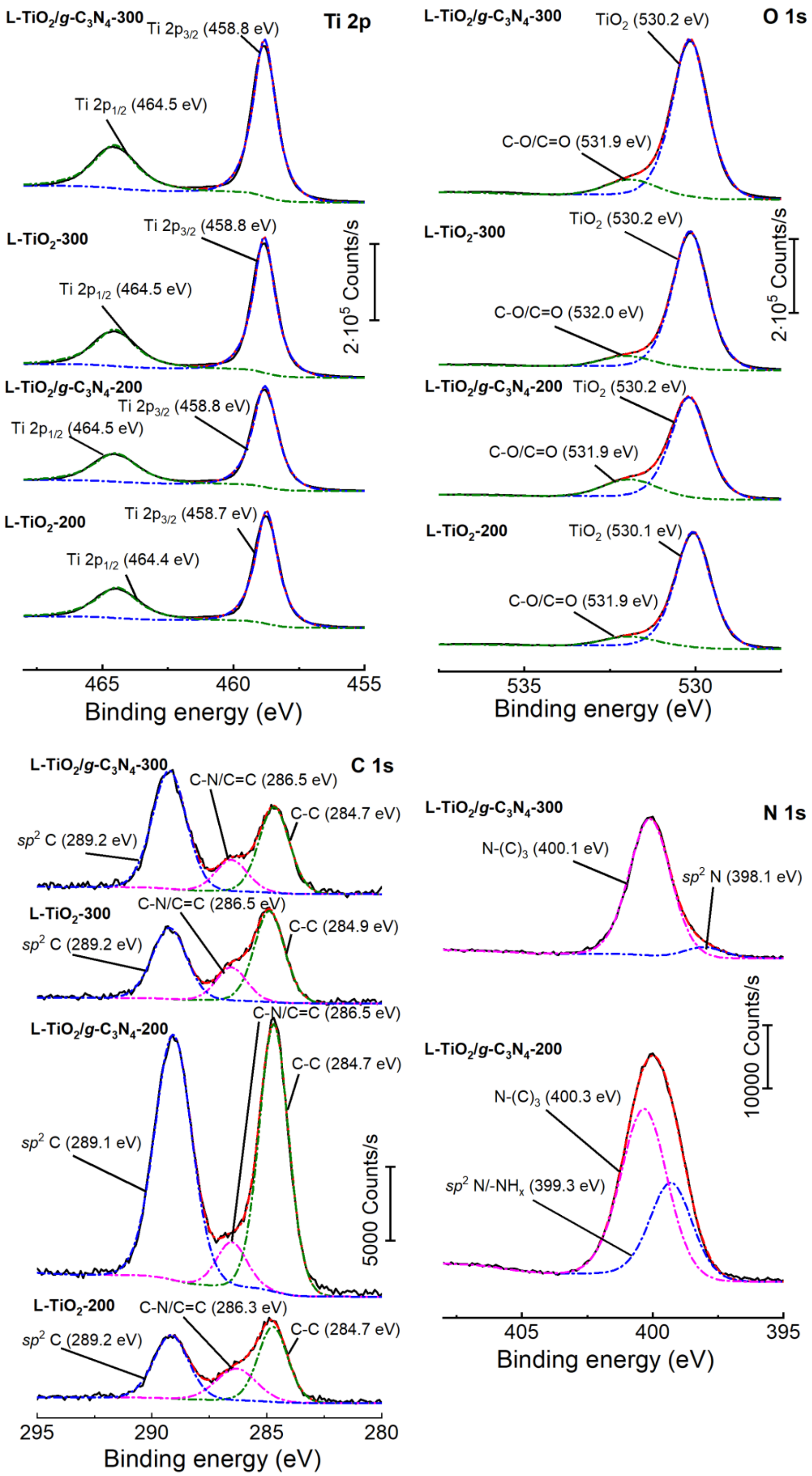


**Fig S4**. High-resolution Ti 2p, O 1s, C 1s, and N 1s spectra for lyophilized $TiO_2$ and $TiO_2$/*g*-$C_3N_4$ samples.

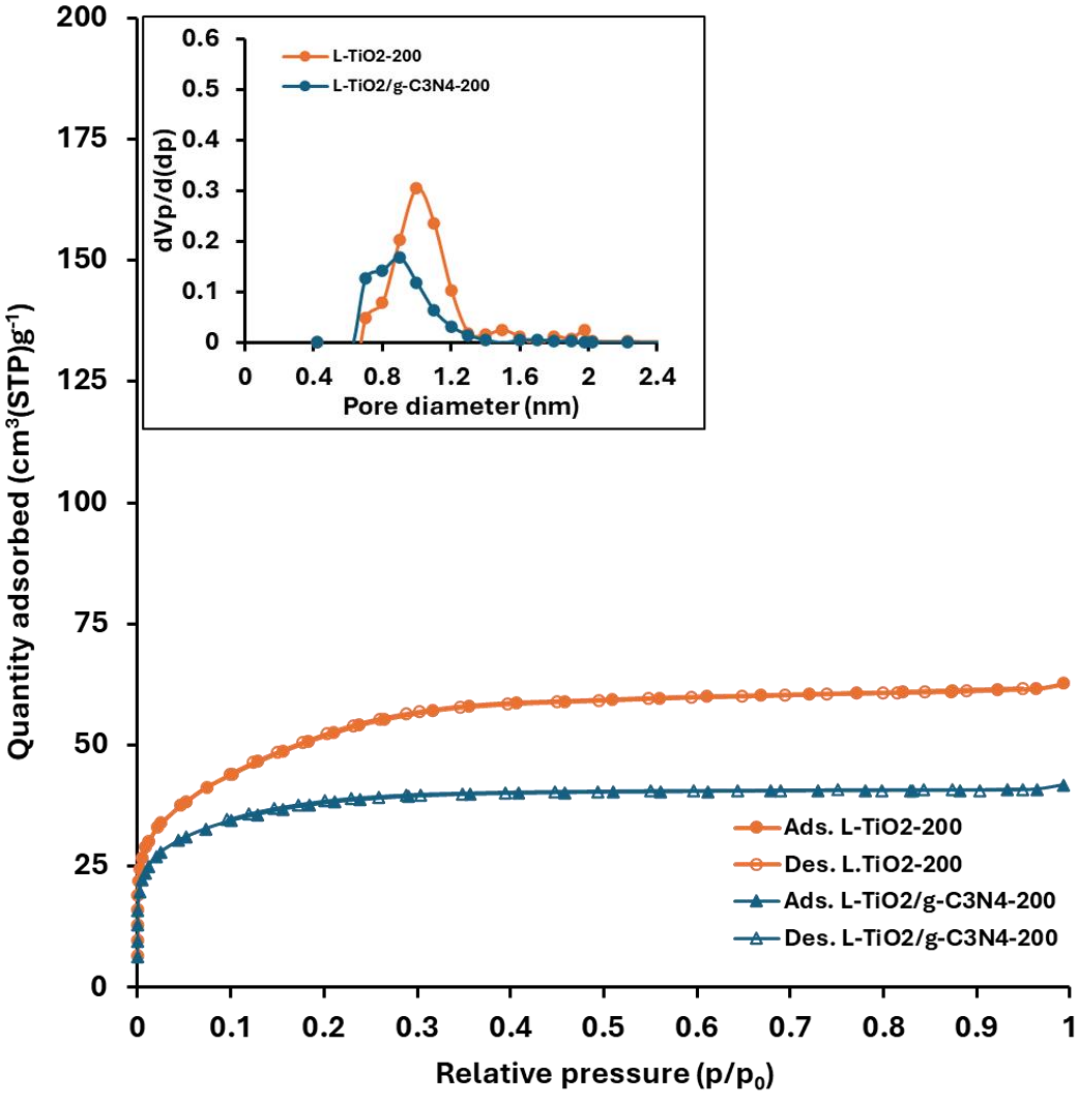


**Fig. S5**. $N_2$ adsorption (filled symbols) and desorption (empty symbols) isotherms and micropore size distributions for samples L-$TiO_2$-200 (red) and L-$TiO_2$/*g*-$C_3N_4$-200 (blue).

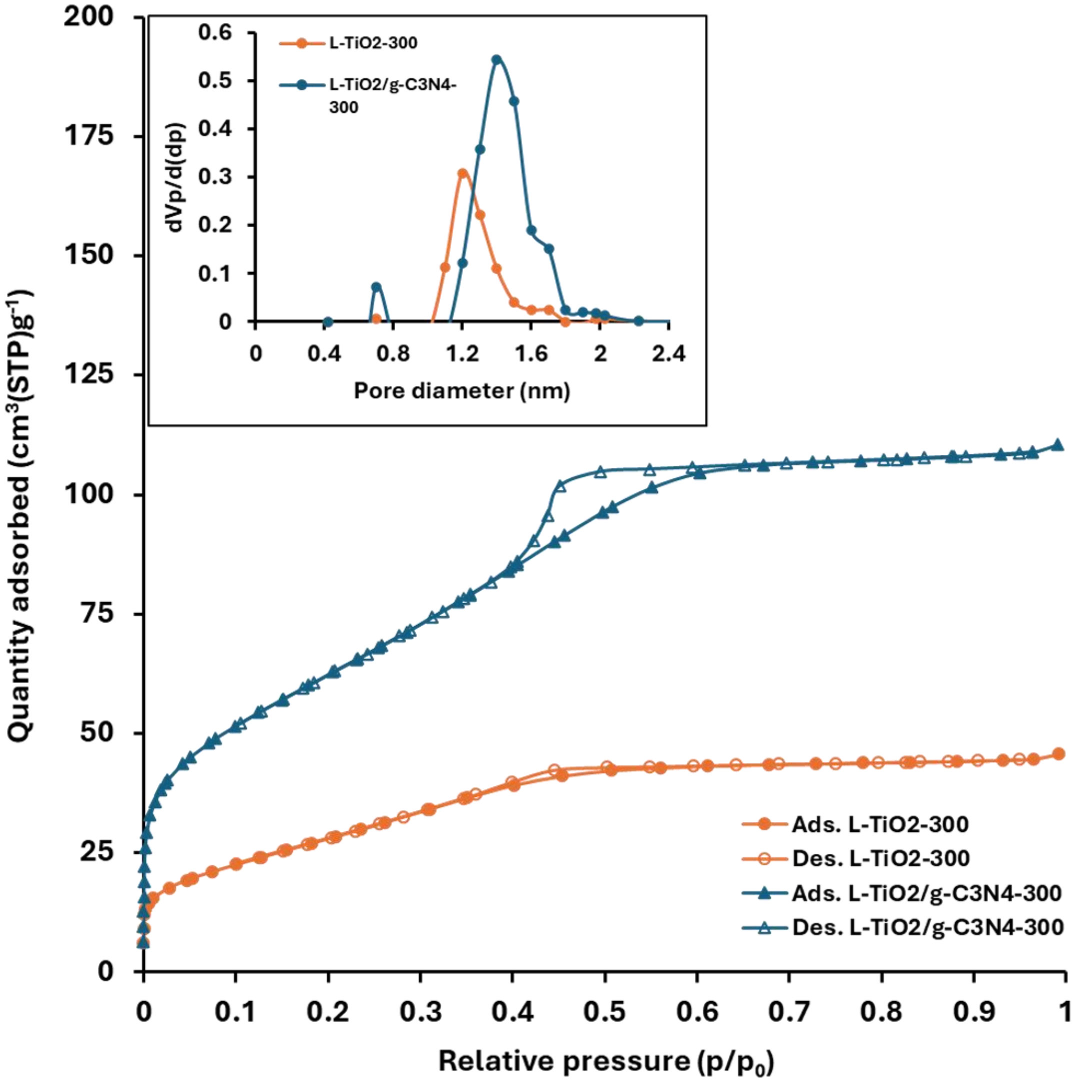


**Fig. S6**. $N_2$ adsorption (filled symbols) and desorption (empty symbols) isotherms and micropore size distributions for samples L-$TiO_2$-300 (red) and L-$TiO_2$/*g*-$C_3N_4$-300 (blue).

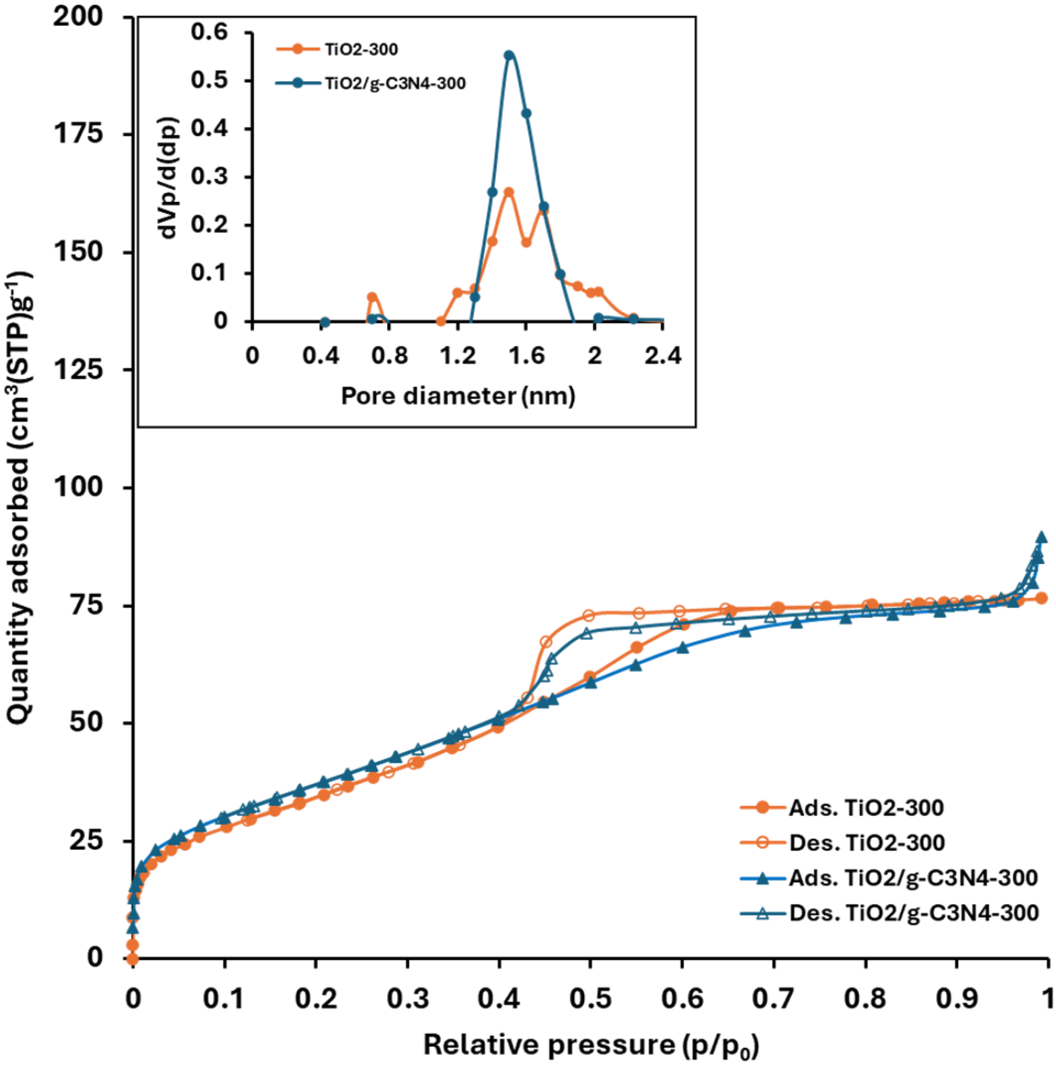


**Fig. S7**. $N_2$ adsorption (filled symbols) and desorption (empty symbols) isotherms and micropore size distributions for samples $TiO_2$-300 (red) and $TiO_2$/*g*-$C_3N_4$-300 (blue).
